# Noncontact Meibography in Patients with Keratoconus

**DOI:** 10.1155/2019/2965872

**Published:** 2019-06-02

**Authors:** Engy Mohamed Mostafa, Marwa Mahmoud Abdellah, Ashraf Mostafa Elhawary, Amr Mounir

**Affiliations:** Ophthalmology Department, Faculty of Medicine, Sohag University, Sohag, Egypt

## Abstract

**Purpose:**

To examine the morphological changes in the meibomian glands of patients with keratoconus as well as to study the relationship between these changes in the morphology and several tear film parameters.

**Methods:**

Examination of the meibomian gland (MG) of 300 keratoconus patients presenting to the center using infrared noncontact meibography system (Sirius, CSO, Italy) between January 2017—January 2019. 100 eyes of healthy individuals were also enrolled as a control group. Tear breakup time (TBUT) test and Schirmer test II were evaluated. Subjective symptoms were also assessed using Ocular Surface Disease Index (OSDI).

**Results:**

Mean age of keratoconus patients was 19 ± 12 years and 21 ± 14 years in control group. Average TBUT was 4.9 ± 2.1 sec. and average Schirmer test was 5.3 ± 2.2 mm which was significantly lower than control group (*p*=0.05). Meibomian gland dropout in the lower eyelid of the keratoconus group was as follows: grade 0 (no loss of meibomian glands): 100 eyes; grade 1 (gland dropout area <1/3 of the total meibomian glands): 85 eyes; grade 2 (gland dropout area 1/3 to 2/3): 68 eyes; and grade 3 (gland dropout >2/3): 47 eyes.

**Conclusion:**

Keratoconus shows significant meibomian gland dropout and distortion that can be recorded by noncontact meibography. Sirius meibography is a simple, cost-effective method of evaluating meibomian gland dropout as a part of the routine refractive examination.

## 1. Introduction

Meibomian gland dysfunction (MGD) is considered the main cause of dry eye disease, leading to evaporative dry eye. The lipid layer in the tear film is derived mainly from the meibomian glands which are of utmost importance for preserving the ocular surface [1]. Meibomian glands (MG) are sebaceous glands located in the eyelids with increasing number in the upper eyelid [2]. MGD-related dry eye can be diagnosed by indirect tests, such as tear breakup time (TBUT) [3] or by direct methods such as meibography, which is using transillumination or infrared (IR) light to image the MGs [4, 5]. Indirect tests are liable for a certain degree of interobserver or intraobserver error. On the contrary, the direct method gives detailed anatomic data of the meibomian glands [6]. MGD has some slit lamp characteristics as clogging of orifices with failure of expressibility of meibum, telangiectasia and hyperemia around the orifices, and thickening of the inner border of the lid margin [7].

Tapie [8] was the first to describe meibography using transillumination of the everted eyelid followed by many other researchers who used confocal microscopy [9], noncontact infrared meibography, [1] or video meibography [10]. Normal meibomian glands appear as hypoilluminant grape-like clusters. However, the orifices and ducts transmit light and appear hyperillunimant [11].

Carracedo et al. [12] reported that keratoconus (KC) patients suffer greater symptoms of dry eye and greater tear instability. Moreover blepharitis was found to occur more often in keratoconus patients than in healthy individuals. Blepharitis is associated with eye rubbing which is considered one of the mechanical etiological factors in keratoconus [13]. Eye rubbing results in sheer strength reduction and cone deformation which may contribute to disease progression [14].

Our aim was to detect structural damage in meibomian glands via meibography in cases of KC and correlating them with indirect tests as TBUT and Schirmer test along with the Ocular Surface Disease Index (OSDI).

## 2. Materials and Methods

### 2.1. Participants

This study examined the meibomian gland of 300 keratoconus patients presenting to the Sohag Cornea and Refractive Center, Sohag, Egypt, using infrared noncontact meibography software in the Scheimpflug topographer (Sirius, CSO, Italy) between January 2017 and January 2019. Hundred eyes of healthy individuals were also enrolled as a control group. The study was approved by the ethical committee of Sohag University and conducted in compliance with the Helsinki declaration. Informed consent was obtained from all of the patients and normal control participants before clinical assessment.

The KC patients enrolled in this study are as follows: Stage 1 included 157 patients, stage 2 had 100, and stage 3 included 43 patients. Only one eye was tested for each patient, and the more diseased eye was the one included in this study. The control participants were randomly selected from patients attending the outpatient clinic and had no signs or symptoms of dry eye or other ocular inflammation.

Exclusion criteria included any other ophthalmic disorder especially blepharitis or chronic use of eye drops for at least 3 months prior to examination, contact lens wearers, eyes with keratoconus grade 4, and chronic systemic disease. The diagnosis of keratoconus was based on classic corneal biomicroscopic and topographic findings in accordance with the criteria of Rabinowitz and McDonnell [15]. Neither the control nor the KC patients reported wearing contact lenses.

### 2.2. Assessment

#### 2.2.1. Ocular Surface Disease Index (OSDI)

The Questionnaire was administered by the examining physician who translated to the patient the 12-item scoring survey, in which the patient rates his or her own ocular symptoms induced by environmental factors over the past 2–4 weeks. Answers were scored on a scale from 0 to 4, with the total score ranging from 0 to 100 and with higher scores denoting greater disability [16].

#### 2.2.2. Tear Breakup Time (TBUT)

TBUT was measured after fluorescein instillation and was represented by the time elapsed from the last complete eyelid blink until appearance of the first dry spot on the cornea. It was measured 3 times consecutively, and the mean value was taken for analysis.

#### 2.2.3. Schirmer II Test

The test (with anesthesia) was performed to evaluate aqueous production. Dryness was considered if wetting of the filter paper was 10 mm or less 2 min after applying topical anesthetic eye drops [17].

#### 2.2.4. Noncontact Meibography

Noncontact meibography was performed by using the Sirius (CSO, Florence, Italy) corneal topographic device with the Phoenix-Meibography Imaging software module. Patients were positioned in front of the scanner, and their forehead was touching the headrest. Only the upper eyelid was evaluated as Dogan et al. reported that it showed better interexaminer agreement as regards grading [18]. Also, upper eyelid MGs outnumber the lower eyelid MGs and are longer in length [19].

The MGs that did not transvere the total tarsal plate were indicated as a “dropout.” The Phoenix software gave the measurements of the dropout by percentage, as well as grouped the dropout by a scale within the area, which was highlighted by the users' free-hand tool: grade 0, no loss at all; grade 1, ≤25%; grade 2, 26%–50%; grade 3, 51%–75%; and grade 4, greater than 75% [6].

#### 2.2.5. Meibograde System

The meibograde system was developed and validated by Call et al. [20]. This system involves gland distortion which is an abnormal gland to tarsus ratio, tortuous glands, and/or discordant patterning depending on previously studied histopathological changes [21–23]. Gland shortening refers to glands not extending from the eyelid margin to the opposite edge of the tarsal plate. Each category was graded from 0 to 3 based on the extent of eyelid involvement: grade 0, no significant eyelid involvement; grade 1, less than 33% involved; grade 2, 33% to 66% involved; and grade 3, more than 66% involved. Then, a maximal score of 9 represented complete gland dropout in the lid [20].

## 3. Statistical Analysis

It was performed by the Statistical Package for the Social Sciences version 17.0 (SPSS Inc, Chicago, Illinois, USA). Normality of the data distribution was tested using the Kolmogorov–Smirnov test. The student test was used to compare gender differences between KC patients and control patients. The Mann–Whitney test was used to determine age and the examination (OSDI, TBUT, and Schirmer test meiboscore) differences among KC patients in different groups and control subjects. ANOVA test was used to compare multiple findings in multiple stages of KC. Spearman correlation was used for detecting correlation between the meiboscore and the other continuous variables. These correlations were considered strong if they were >0.80, moderately strong if they were between 0.5 and 0.8, fair if they were within the range of 0.3 and 0.5, and poor if they were <0.30 [24]. A value less than 0.05 was considered to be statistically significant.

## 4. Results

Table 1 shows the difference between the KC and control group in demographic data as well as in clinical tests. KC group and the control group were age and sex matched with no statistical difference. The TBUT and Schirmer test indicated statistically significant differences between both groups with the lower values belonging to the KC group. The OSDI was significantly higher in the KC group than that in the control group. On the contrary, there was no statistical difference in meibography between the KC group and the controls. On stratifying the KC groups according to their stage, there was only significant difference in the OSDI (Table 2).

**Figure 1:**
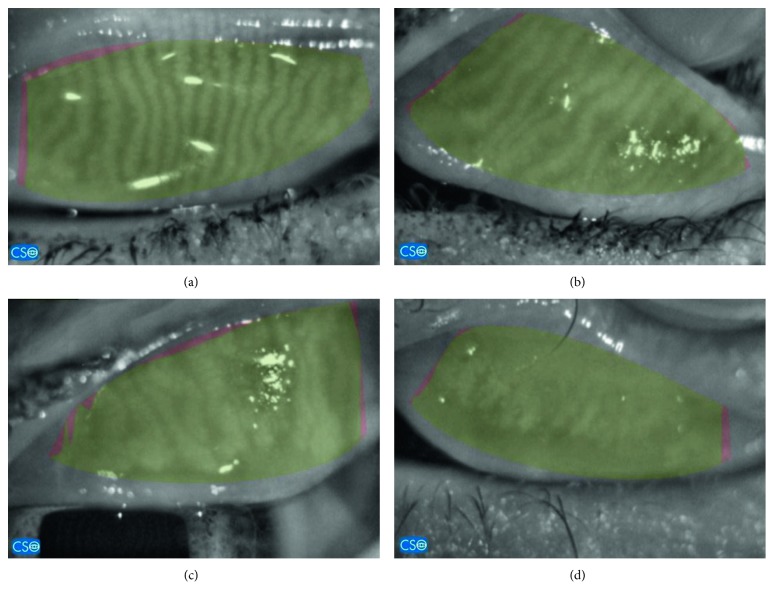
(a) Normal meibomian glands with no distortion nor dropout. (b) Grade 1 with dilatation and tortuosity of the MG. (c) Grade 2: dropout of MG along with gland distortion. (d) Grade 3: MG does not traverse the total tarsal with mottling of details.

Meibomian gland dropout in the upper eyelid of the whole KC group according to the Phoenix software was grade 0 (no loss of meibomian glands): 100 eyes; grade 1: 142 eyes; and grade 2: 58 eyes; grades 3 and 4: 0 eyes (Figure 1).  Table 3 shows the number of patients showing each type of characteristic gland abnormality as well as its divided scores. There was statistical difference between the different groups of KC and the control group in the gland dropout with a total score approaching significance. While on comparing the whole KC groups against the control group, there was significance in all characteristics. KC stage 3 showed significant difference from KC stage 1 in gland distortion and shortening as well as the total score. There was no difference between all stages in gland dropout.

Meiboscore correlated significantly with age, sex, KC stage, Schirmer test, and TBUT (ranging between fair and moderate correlation) (Table 4). Yet, it did not have any significant correlation with the OSDI.  Table 5 shows correlation between the shortening, distortion, and dropout of MG with other parameters: there was fair correlation with clinical significance between all gland characteristics and KC staging as well as TBUT and Schirmer test.

## 5. Discussion

Our results of meibography imaging showed no difference between the keratoconus group and the control group. There was only significant difference in gland distortion between different stages of KC. Yet the meiboscore correlated well with the KC staging, TBUT, and Schirmer test. And as expected, all clinical testing of dry eye showed clinical difference between the KC patients and the controls. There have always been indirect methods of evaluating MGD such as TBUT and tear osmolarity. Despite the fact that they are objective, results can vary due to interobserver and intraobserver differences [25]. The direct imaging of meibomian gland can offer an anatomical analysis that can contribute to the scope of diagnosis and treatment as well [25].

Keratoconus shows higher dropout in MG when compared to the control group despite the fact that there was no significant difference in the meiboscore. This might be attributed to the young age group of the KC patients. In further studies, evaluation of an older group of KC patients would help elucidate the progress of the MG dysfunction. There was no correlation between the OSDI sand the meibography grading. Ngo et al. [26] reported that dropout scores based on the IR images for MGs did not correlate with clinical signs as well. Blackie et al. mentioned that nonchronic blepharitis with no visible inflammation can cause evaporative dry eye which might interpret the lack of correlation between meibograding and OSDI [27].

Our results show that OSDI scores were much higher in the KC group compared to those in control which relates to the results by Dienes et al. [28].

The importance of detecting MGD in cases of KC patients lies in the presence of different lines of treatments that should be chosen depending on the diagnosis to guarantee an optimum response. The appropriate treatment would work on reducing the burning sensation, irritation, tearing, photophobia, blurred vision, and red eyes related to blepharitis thus decreasing patients' tendency to rub their eyes which would eventually improve the quality of vision. On one hand, obstructive MGD with the dropout of acini would benefit from lipid-containing eye drops to improve the stability of the tear film [29–31]. On the other hand, in advanced cases of obstructive MGD that show progressive loss of acini, treatment may involve eyelid hygiene [32] and warm compresses to improve the secretion function [33].

Different technologies were used for meibography [34] such as infrared meibography, [1] confocal meibography, [9] and optical coherence meibography [35]. For comparing the technology, we used the confocal technique: the latter has the disadvantage of being a contact method that can result in patient discomfort, [9] while the optical coherence method shows a relatively difficult interpretation as it requires testing at the same area for consequent measurement [35]. Arita et al. [36] demonstrated diagnostic cutoff values for the meiboscore in combination with symptoms and lid margin abnormalities with a sensitivity of 84.9% and specificity of 96.7% for the diagnosis of MGD.

We are aware that this study focuses on the anatomic details of the MG rather the function of the meibum or its chemical composition which warrants further studies. Yet the indirect tests and the questionnaire were an attempt to correlate the dropout of MG and its effect on the function of the tear film.

In general, meibography provides a feasible method of recording and documenting the MGs for better diagnosis of its dysfunction in various diseases and its severity. It should be taken into account that meibography should be used in context of clinical findings and symptoms. Sirius meibography is a simple, noncontact, cost-effective method of evaluating meibomian gland dropout as a part of the routine refractive examination. Accessible screening of MGs dropout and distortion in KC patients allows for better management of dry eye diseases in these patients. Effective management of dry eye disease makes it possible to decrease eye rubbing and thus reduce the mechanical stress on the already vulnerable corneas.

## Figures and Tables

**Table 1:** Clinical findings of both groups.

Mean ± SD	Keratoconus group (*n* = 300)	Control group (*n* = 100)	*p* value
Sex (M/F)	133/167	42/58	0.23
Age	19 ± 12	21 ± 14	0.25
OSDI score	32.12 ± 14.2	12.2 ± 6.5	0.032
TBUT (sec.)	4.9 ± 2.1	8.3 ± 3.3	0.02
Schirmer test (mm)	5.3 ± 2.2	9.4 ± 3.4	0.05
Total meiboscore	1.36 ± 1.2	1.02 ± 1.1	0.06

OSDI: Ocular Surface Disease Index; TBUT: tear breakup time.

**Table 2:** Clinical findings in different keratoconus stages.

	KC 1	KC 2	KC 3	*p* value
OSDI	30.1	32.5	33.8	0.027
TBUT (sec.)	5.2	5.1	4.6	0.79
Schirmer II test (mm)	5.9	5.3	4.8	0.07
Meiboscore	2.1	2.4	2.6	0.32

OSDI: Ocular Surface Disease Index; TBUT: tear breakup time.

**Table 3:** Meibomian gland characteristics in all groups.

Mean ± SD (*n*)	KC 1 (*n* = 157)	KC 2 (*n* = 100)	KC 3 (*n* = 43)	Control (*n* = 100)	*P*_0_	*P*_1_	*P*_2_	*P*_3_
Gland distortion	0.22 ± 0.11 (30)	0.21 ± 0.12 (21)	0.18 ± 0.11 (8)	0.21 ± 0.14 (18)	0.423	0.09	0.21	0.04
Gland shortening	0.31 ± 0.12 (44)	0.35 ± 0.18 (18)	0.36 ± 0.15 (8)	0.33 ± 0.13 (19)	0.751	0.087	0.088	0.023
Gland dropout	0.71 ± 0.25 (26)	0.73 ± 0.28 (12)	0.79 ± 0.33 (17)	0.51 ± 0.23 (11)	0.002	0.75	0.44	0.56
Total score	2.1	2.4	2.6	1.5	0.06	0.05	0.21	0.025

KC: keratoconus. *n*: number of patients; SD: standard deviation. *P*_0_ value between the four groups by ANOVA test. *P*_1_ value: KC 1 vs KC 2. *P*_2_ value: KC 2 vs KC 3. *P*_3_ value: KC 1 vs KC 3.

**Table 4:** Correlation between the meiboscore and other factors in KC patients.

	Meiboscore	
	*r*	*p*
Age	0.421	0.006
Sex	0.509	0.03
KC stage	0.621	0.05
OSDI	0.162	0.72
TBUT	0.320	0.02
Schirmer II test	0.499	0.032

KC: keratoconus; OSDI: Ocular Surface Disease Index; TBUT: tear breakup time.

**Table 5:** Correlation between the meibomian gland characteristics in meibography and other factors in KC patients.

	Gland distortion		Gland shortening		Gland dropout	
	*r*	*p*	*r*	*p*	*r*	*p*
Age	0.12	0.92	0.16	0.87	0.22	0.82
KC stage	0.21	0.45	0.42	0.64	0.22	0.45
OSDI	0.23	0.34	0.33	0.43	0.31	0.53
TBUT	0.22	0.05	0.18	0.04	0.24	0.05
Schirmer test	0.21	0.03	0.19	0.032	0.22	0.05

KC: keratoconus; OSDI: Ocular Surface Disease Index; TBUT: tear breakup time.

## Data Availability

The data used to support the findings of this study are available from the corresponding author upon request.
